# Supplementary material for: The interactome of intact mitochondria by cross-linking mass spectrometry provides evidence for coexisting respiratory supercomplexes
Source: Mol Cell Proteomics. 2017 Dec 8;17(2):216–32. doi: 10.1074/mcp.RA117.000470 (PMC5795388; doi:10.1074/mcp.RA117.000470)
Supplement: Supplemental Data [file supp_RA117.000470_133922_0_supp_23978_fzffwf.zip › spectra_annotation/mito_spectra_annotation/452-1-1-1-9-1.pdf]

LKEVGRI
SDRPELTGAKVVVSGGR

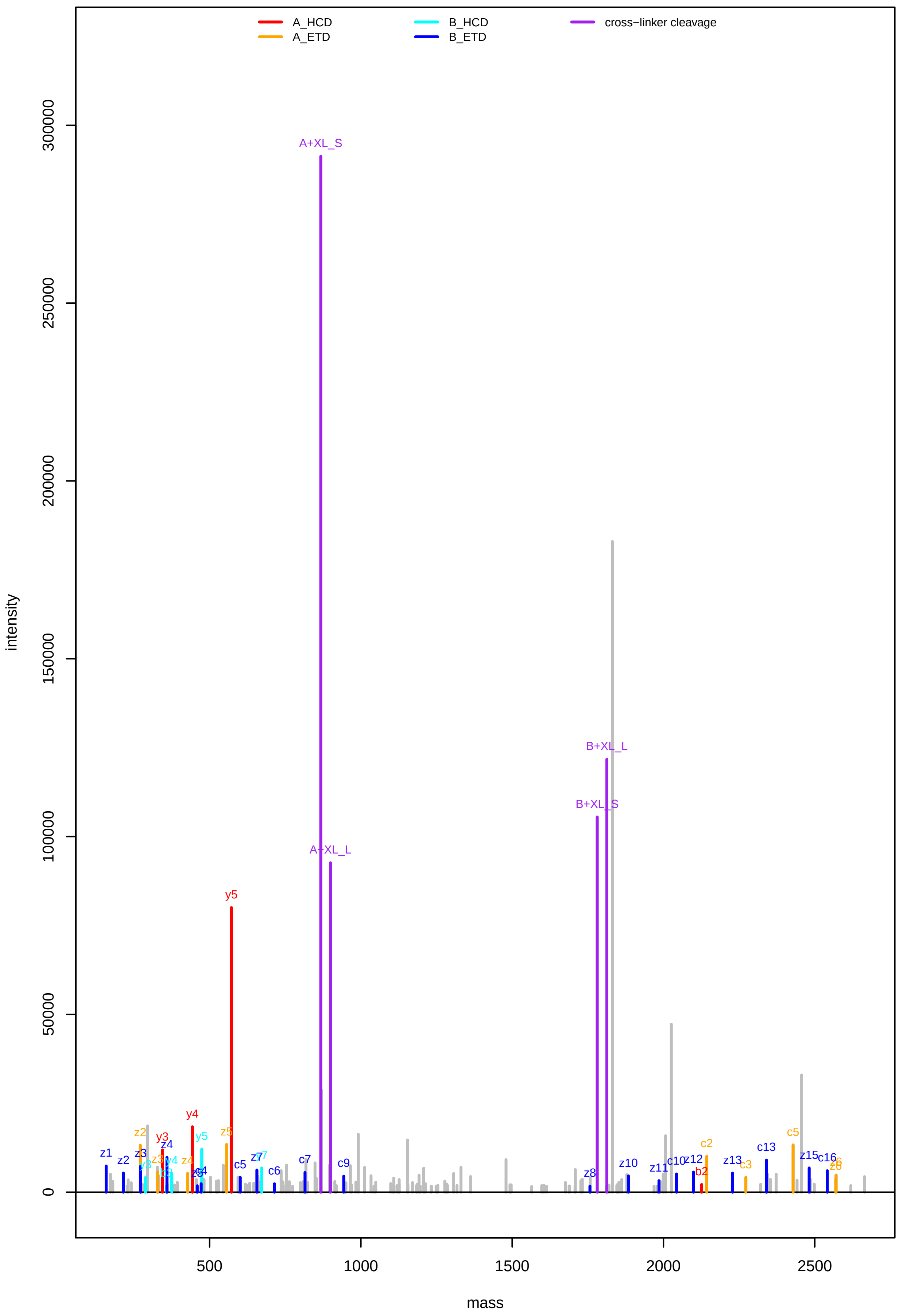

LKEVGRI+XL_S

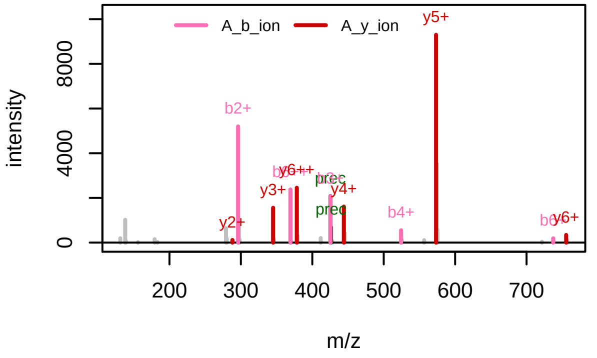

LKEVGRI+XL_L

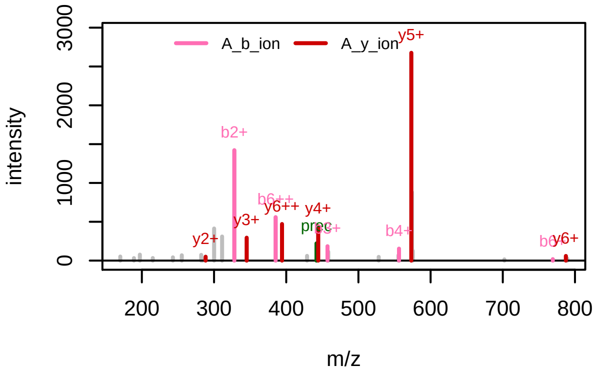

SDRPELTGAKVVVSGGR+XL_S

SDRPELTGAKVVVSGGR+XL_L
